# Evaluating the Effect of an Essential Oil Blend on the Growth and Fitness of Gram-Positive and Gram-Negative Bacteria

**DOI:** 10.3390/biology14040437

**Published:** 2025-04-18

**Authors:** Kelechi A. Ike, Paul C. Omaliko, Mizpha C. Fernander, Brittany M. Sanders, James M. Enikuomehin, Joel O. Alabi, Oludotun O. Adelusi, Ahmed E. Kholif, Misty D. Thomas, Uchenna Y. Anele

**Affiliations:** 1 Department of Animal Sciences, North Carolina Agricultural and Technical State University, Greensboro, NC 27411, USA; kaike@aggies.ncat.edu (K.A.I.); pcomaliko@aggies.ncat.edu (P.C.O.); jmenikuomehin@aggies.ncat.edu (J.M.E.); joalabi@aggies.ncat.edu (J.O.A.); ooadelusi@aggies.ncat.edu (O.O.A.); aekholif@ncat.edu (A.E.K.); 2 Department of Biology, North Carolina Agricultural and Technical State University, Greensboro, NC 27411, USA; mcfernan@ncat.edu (M.C.F.); brsander@aggies.ncat.edu (B.M.S.); mthomas1@ncat.edu (M.D.T.)

**Keywords:** antimicrobial activity, bacterial growth, MIC, pathogens, growth kinetics, fitness cost

## Abstract

The increasing prevalence of antibiotic-resistant bacteria has necessitated the exploration of alternative antimicrobial agents, particularly natural products like essential oils. This study investigated the antibacterial potential of a unique blend of four essential oils (EOB) across a gradient of concentrations (0.1 to 50%) against Gram-positive and Gram-negative bacteria using an adapted broth microdilution method, minimum inhibitory concentrations (MICs), and 24-h growth assays. The Gram-positive bacteria were *Staphylococcus epidermidis* and *Bacillus subtilis*, while the Gram-negative bacteria were *Escherichia coli* and *Klebsiella aerogenes*. The results demonstrated that the EOB exerted a concentration-dependent inhibitory effect on bacterial growth, with MICs determined at 25% for all the species tested. Growth curve analysis revealed that lower concentrations of the EOB (0.1 to 0.78%) allowed for normal bacterial proliferation, while at intermediate concentrations (1.56 to 3.13%), inconsistent trends in growth were exhibited. At higher concentrations (25 and 50%), the EOB effectively halted bacterial growth, as indicated by flat growth curves. The increase in the lag phase and the decrease in the growth rate at a sub-MIC concentration (12.5%) suggest a significant effect on bacterial adaptation and survival. Relative fitness analyses further highlighted the inhibitory effects of higher essential oil concentrations. *S. epidermidis* and *E. coli* had a significant (*p* < 0.05) reduction in fitness starting from the 6.25% concentration, while the other two species experienced a significant (*p* < 0.001) reduction in relative fitness from a concentration of 12.5%. These findings underscore the potential of this EOB as an effective antimicrobial agent, particularly in the context of rising antibiotic resistance. Furthermore, the study suggests that the EOB used in the present study could be integrated into therapeutic strategies as a natural alternative or adjunct to traditional antibiotics, offering a promising avenue for combating resistant bacterial strains.

## Simple Summary

The rise in antibiotic-resistant bacteria has created an urgent need for alternative antimicrobial solutions. This study explores the effects of a unique blend of essential oils on the growth and survival of different bacterial species, including both Gram-positive and Gram-negative bacteria. By testing a range of concentrations, we found that higher concentrations of the essential oil blend significantly inhibited bacterial growth, while lower concentrations had little to no effect. The study also assessed bacterial fitness, revealing that increasing essential oil concentrations reduced the ability of bacteria to grow and survive. These findings highlight the potential of this essential oil blend as a natural alternative to antibiotics. If further validated, it could be integrated into antimicrobial treatments, contributing to efforts to combat antibiotic resistance in both medical and agricultural settings.

## 1. Introduction

The management of pathogenic infections has increasingly captured public interest. However, the emergence of antibiotic resistance has significantly complicated the treatment of pathogenic bacterial infections and has increased awareness of the discovery of revolutionary bioactive substances that can inhibit a wide range of organisms [1]. *Staphylococcus epidermidis*, *Escherichia coli*, *Klebsiella aerogenes*, and *Bacillus subtilis* are clinically relevant pathogens that are responsible for various infections in medical and community settings. *S. epidermidis* frequently causes healthcare-associated infections such as catheter-related bloodstream infections, prosthetic joint infections, and endocarditis, which are primarily linked to biofilm formation on implanted medical devices [2]. *E. coli* is well known for its pathogenic roles in gastrointestinal infections, urinary tract infections, neonatal meningitis, and septicemia, making it a significant concern in both clinical and public health contexts [3]. Similarly, *K. aerogenes* causes nosocomial infections, including urinary tract, respiratory tract, bloodstream, and wound infections, highlighting its versatility as an opportunistic pathogen with increasing antibiotic resistance [4]. *B. subtilis* is generally considered non-pathogenic but can occasionally cause opportunistic infections, including bacteremia and endocarditis, particularly in immunocompromised hosts [5]. Treating these bacterial species is crucial for both human and animal health industries, as they contribute to a wide range of opportunistic and hospital-acquired infections, are linked to antimicrobial resistance, and significantly impact public health and clinical outcomes. While antibiotics have been effective in mitigating their adverse effects in livestock, regulatory policies on antibiotic use in animal feed have recently undergone changes and continue to evolve. This has driven interest in bioactive secondary metabolites as alternative performance enhancers [6]. Therefore, exploring safe and natural alternatives, such as essential oils, is highly recommended.

Essential oils (EOs) are excellent sources of bioactive compounds possessing antibacterial and antioxidative effects and have become of great interest in the pharmaceutical, food, and livestock industries [7]. Incorporating EOs, such as oregano oil, into the diets of neonatal calves has been shown to improve their immune system, leading to enhanced feed efficiency and potentially reducing the need for conventional medications, thereby lowering treatment costs [8]. Using EOs as an antimicrobial intervention offers the added advantage of potentially reducing the potential for antimicrobial resistance development, and this can be attributed to their complex chemical composition, which encompasses numerous antibacterial pathways [9]. Mittal et al. [10] reported that EOs can interact with various cellular targets and have various effects, including the disruption of the cytoplasmic membrane, inhibition of protein synthesis, and interference with efflux pump activity, among others. Lavender EO (*Lavandula angustifolia*) has been shown to alleviate inflammation caused by *Staphylococcus aureus* infection by enhancing phagocytosis and reducing the production of pro-inflammatory cytokine production [11].

Prior research has examined single essential oils at limited concentrations and focused on endpoints such as the minimum inhibitory concentration (MIC), providing little insight into how sub-inhibitory doses or complex EO mixtures affect bacterial growth dynamics and fitness [12,13,14]. Essential oil combinations can exhibit synergistic effects, enhancing their antimicrobial efficacy beyond what is observed with individual components. A commercial EO product (Olbas^®^ Tropfen; Deutsche Olbas, GmbH, Magstadt, Germany) containing peppermint, eucalyptus, cajuput, and juniper has been shown to be effective against a variety of pathogens, including methicillin-resistant *S. aureus* (MRSA) and vancomycin-resistant *Enterococcus* and is an indication that the product could be useful in the treatment of simple cutaneous and respiratory infections [15]

Therefore, this preliminary study evaluated a unique novel blend of five EOs (garlic, lemongrass, cumin, lavender, and nutmeg in the ratio 4:2:2:1:1, respectively) against representative Gram-positive and Gram-negative bacteria across a broad concentration gradient (0.1–50%). While many previous studies have investigated individual essential oils, this unique blend comprises monoterpenes, phenolics, and organosulfur compounds, potentially resulting in a multifaceted antimicrobial agent. Instead of relying solely on endpoint assays, 24-h bacterial growth curves were monitored, and the growth kinetics were modeled to quantify changes in growth parameters and relative fitness under varying EO concentrations. This comprehensive approach provides novel insight into the concentration-dependent effects of the EO blend (EOB), revealing sub-lethal impacts on bacterial proliferation that are often overlooked, underscoring the blend’s potential as a natural antimicrobial alternative amid rising antibiotic resistance. By evaluating its efficacy under controlled conditions, we aim to establish a scientific basis for its application in combating bacterial pathogens while addressing growing concerns over antibiotic resistance and restrictions.

## 2. Materials and Methods

### 2.1. Experimental Site

This experiment was carried out in the Departments of Biology and Animal Sciences at the North Carolina Agricultural and Technical State University, Greensboro, NC, USA.

### 2.2. Bacteria

Two Gram-positive (*B. subtilis* and *S. epidermidis*) and two Gram-negative (*E. coli* and *K. aerogenes*) bacterial strains were used. The bacteria were obtained from the American Type Culture Collection (ATCC), Manassas, VA, USA. They were stored at −80 °C in Lysogeny Broth (LB) medium with glycerol.

### 2.3. Preparation of the Essential Oil Working Solution

The EOB used in this study has been used in a previous study [16]. The analysis of the volatile compounds in the EOB was performed using an Agilent 7890 GC system equipped with a 5977B GC/MSD and a 7693 autosampler (Agilent, Santa Clara, CA, USA). A 2 µL aliquot of the sample was injected with a split ratio of 1:50. The oven temperature was initially held at 50 °C for 1 min, followed by a ramp of 8 °C/min to 220 °C, where it was maintained for 10 min, resulting in a total run time of 32.25 min. The mass spectrometer operated in scan mode, with a mass range of 50–500 amu and an electron ionization voltage of 70 eV.

Due to the hydrophobic nature of the EOs, a broth microdilution technique was employed, following the CLSI 2018 standard methodology (document M07) [17] and drawing from previous similar studies [18,19]. This method was used to obtain the hydrosoluble constituents of the EOB, which confer the antibacterial activity. To achieve this, equal volumes of the EOB and sterile water were combined in 15 mL sterile centrifuge tubes, and using an orbital plate mixer, the mixture was carefully but thoroughly mixed overnight. The bottom aqueous phase was recovered and utilized as the EO working solution.

### 2.4. Determination of the Minimum Inhibitory Concentrations and the Growth Curve Assay

The first step involved inoculating archived bacterial glycerol stocks into LB media (Sigma-Aldrich, St. Louis, MO, USA) and incubating them overnight at 37 °C with continuous shaking at 200 rpm. Upon retrieval from the incubator, the overnight cultures were diluted to an absorbance (ABS) of 0.05 at 600 nm for normalization.

**Figure 1:**
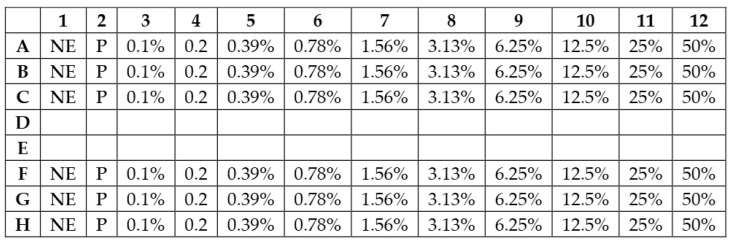
Plate design of the experiment. NE: Negative control, P: Positive control.

The minimum inhibitory concentrations (MIC) and 24 h growth assays were conducted for the bacterial species in 96-well plates (eight rows marked from A–H and 12 columns marked from 1–12) (Figure 1). Two 96-well plates (Thermo Fisher Scientific, Waltham, MA, USA) were used, with two bacterial species assigned to each plate. Each species was tested in triplicate. Rows A, B, and C contained one bacterial species while rows F, G, and H contained another. The middle two rows, D and E, were left blank for demarcation and to avoid cross-contamination. Exactly 200 µL of EOB was introduced into column 12. One hundred microliters of sterile water was distributed in columns 1 to 11. Using a multichannel pipette, 100 µL of the EO from the initial column (column 12) underwent sequential mixing with the sterile water up to column 3, when the last one 100 µL was discarded; this process resulted in two-fold serial dilutions. The final concentrations of the EOB were therefore 50, 25, 12.5, 6.25, 3.13, 1.56, 0.78, 0.39, 0.20, and 0.10%, *v*/*v* in the final volume, which is equivalent to 500, 250, 125, 64, 32, 16, 8, 4, 2, and 1 µL/mL. There was no EOB in column 2, which served as the positive control, while column 1 had neither EOB nor the bacterial stock (only LB broth and sterile water), making it the negative control.

The plates were sealed with an optically clear MicroAmp Optical Adhesive film (Thermo Fisher Scientific^®^, Waltham, MA, USA) and incubated for 24 h at 37 °C with shaking in a Varioskan Lux 96-well plate reader (Thermo Fisher Scientific^®^, Waltham, MA, USA). Measurements were taken at ABS of 600 nm at 0, 2, 4, 6, 8, 10, 12, 16, 20 and 24 h. The absorbance data were normalized using the negative controls, with the mean absorbance values subtracted from each replicate population at each concentration. The results were subsequently plotted to generate 24 h growth curves using GraphPad Prism version 8.0.0 (GraphPad Software, San Diego, CA, USA). The bacterial growth metrics (i.e., carrying capacity, growth rate, time at midpoint of the growth curve, generation time, and area under the empirical and logistic curve) were generated from the growth curve data using the GrowthCurver package in R (version 0.3.1). The package was developed by Sprouffske and Wagner [20] and is available on the Comprehensive R Archive Network (CRAN). The source code is available under the GNU General Public License and can be obtained from Github (San Francisco, CA, USA). Relative fitness (RF) values indicate the bacterial population’s ability to survive and the proliferate changes in response to increasing concentrations of the EOB. To calculate relative fitness, the reproductive rate of each population was divided by the reproductive rate of other genotypes in the population at the same timepoints. Analysis of variance (ANOVA) was performed using GraphPad Prism to calculate statistical differences, with significance defined as *p* < 0.05. Multiple comparisons were performed between each concentration and the positive control.

## 3. Results

### 3.1. Bioactive Compounds

The GC-MS analysis of the EOB identified a complex mixture of bioactive volatile compounds (Table 1). Generally, beta-Phellandrene (20.30%), beta-Myrcene (12.13%), Terpinen-4-ol (10.53%), Geranyl formate (9.36%), Bicyclo[2.2.1]heptane, 2,2-dimethyl-3-methylene-, (1S)- (6.77%), p-Cymen-7-ol (6.71%), 1,3-Cyclohexadiene, 1-methyl-4-(1-methylethyl)- (6.45%), Eucalyptol (6.24%), 4-Nonanone (5.18%), and 5-Hepten-2-one, 6-methyl- (3.00%) were the predominant volatile components. Together, these ten compounds represented approximately 87% of the total composition. Nonetheless, minor components were also present, and these may play a significant role in the observed biological activity and antibacterial effects.

### 3.2. Minimum Inhibitory Concentrations

**Figure 2:**
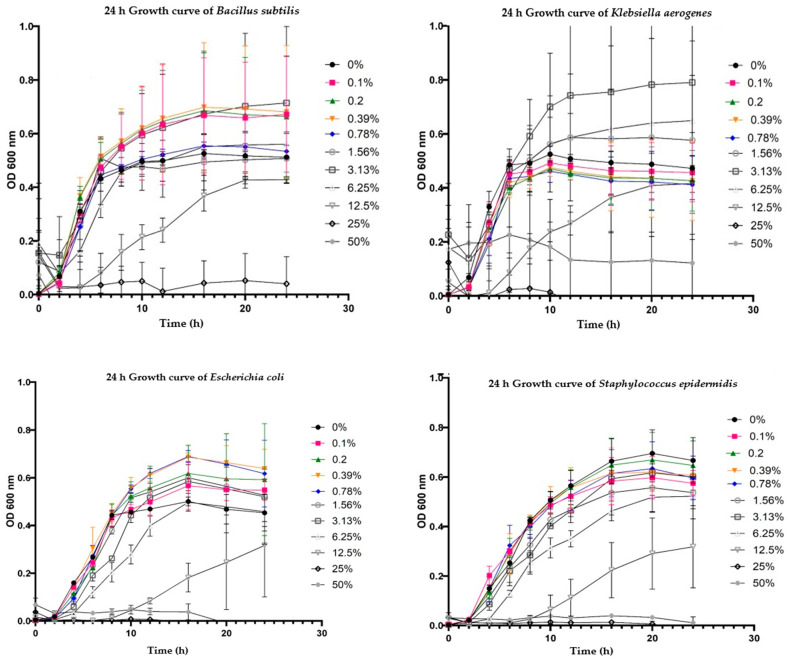
Effect of the different EOB concentrations on the growth curve of *S. epidermidis*, *E. coli*, *K. aerogenes*, and *B. subtilis*.

The MIC values were at 25% concentration across all the bacterial species investigated. The growth curves for the bacteria are presented in Figure 2. The lag phase across all the bacteria was generally within the first 2 h, where the absorbance values were low across all the concentrations except for the sub-MIC concentration, where the lag phase extended up to 4 h. Between 4 and 10 h, a significant increase in absorbance was observed, particularly in the control treatment (0% EOB concentration) and at lower concentrations of the EOB (0.1 to 0.78% EOB). This suggests rapid bacterial growth during this period. In contrast, the bacteria treated with intermediate concentrations (1.56 to 3.13% EOB) exhibited inconsistent trends in absorbance, while the higher concentrations had a slower increase in absorbance, indicating that the EOB caused some inhibition of bacterial growth. At 25 and 50% EOB, the curves were flat across all the bacterial species, indicating little to no growth. The sub-MIC concentration (12.5% EOB) showed a notable weak increase in absorbance across all the bacterial species. Overall, the absorbance values generally plateaued after 10 h.

### 3.3. Relative Fitness

The data also includes *p*-values adjusted for multiple comparisons to assess the statistical significance of these changes (Table 2).

#### 3.3.1. *S. epidermidis*

Table 2 shows the relative fitness of *S. epidermidis* at various concentrations of the EOB compared with the positive control (0% EOB concentration). The relative fitness values ranged from approximately RF = 0.85 at the lower concentrations to as low as 0.05 at higher concentrations. At lower concentrations (0.1 to 3.13% EOB), the relative fitness of *Staphylococcus* remained relatively high (between RF = 0.85 and 0.67), with no statistically significant difference compared with the control (adjusted *p*-values > 0.9999 for most comparisons). This indicates that the EOB at these concentrations did not significantly affect the fitness of the bacteria. At the 6.25% EOB concentration, the relative fitness dropped to approximately RF = 0.51, with a highly significant adjusted *p*-value (<0.0001). This suggests a substantial impact of the EOB on bacterial fitness. At 12.5% EOB, the fitness further decreased to RF = 0.21, also with a highly significant *p*-value (<0.0001). This marked decrease in fitness was observed at higher concentrations (25 and 50% EOB), where the relative fitness dropped to approximately RF = 0.06 and 0.15, respectively. These results indicate that the EOB exerted a potent inhibitory effect on bacterial fitness at higher concentrations, with statistically significant adjusted *p*-values (<0.0001).

#### 3.3.2. *E. coli*

At lower concentrations (0.1 to 3.13% EOB), the EOB did not produce a statistically significant reduction in *E. coli* relative fitness (Table 2). In fact, the fitness slightly increased, although these changes were not statistically significant (*p*-values > 0.9999 to 0.8759). However, at a concentration of 6.25% EOB, the relative fitness of *E. coli* significantly decreased to RF = 0.48, indicating that this concentration begins to impair bacterial viability or proliferation to a similar extent as that observed for *S. epidermidis*. At higher concentrations (12.5 to 50% EOB), the EOB demonstrated potent antimicrobial activity, as evidenced by the sharp decline in relative fitness. The relative fitness significantly decreased (*p* < 0.0001) to RF = 0.15 at 12.5% and to nearly zero at 25 and 50% EOB, indicating that these concentrations are bactericidal for *E. coli*.

#### 3.3.3. *K. aerogenes*

The relative fitness of *K. aerogenes* at various concentrations of the EOB compared with a positive control (0 EOB concentration) is shown in Table 2. At lower concentrations of the EOB (0.1 to 3.13% EOB), the relative fitness values ranged from approximately RF = 0.62 to 0.84, with none of these reductions being statistically significant (*p*-values ranging from 0.9979 to 0.7450). This suggests that these low concentrations did not have a significant impact on the RF of *K. aerogenes*. At 6.25% EOB, the relative fitness increased slightly to RF = 0.73 compared with the control, but this change was not statistically significant (*p* > 0.9999). This suggests that the EOB at this concentration did not inhibit *K. aerogenes* effectively and may even have slightly stimulated its growth or viability. A significant reduction in fitness was observed at higher concentrations. At 12.5% EOB, the relative fitness dropped to RF = 0.32 (*p* = 0.0007), and at 25% EOB, it further decreased dramatically to RF = 0.07 (*p* < 0.0001). At 50% EOB, the relative fitness was slightly higher (RF = 0.32) than the value recorded for the 25% EOB concentration, but this still represented a significant reduction compared with the control (*p* = 0.0008). These findings indicate that higher concentrations of the EOB have a substantial inhibitory effect on the fitness of *K. aerogenes*.

#### 3.3.4. *B. subtilis*

Table 2 presents the relative fitness of *B. subtilis* at various concentrations of the EOB compared with the positive control. At lower concentrations (0.1 to 3.13%), the relative fitness values ranged from approximately RF = 0.66 to 0.82. Interestingly, the fitness increased slightly at 0.1 and 0.2% EOB, but these changes were not statistically significant (*p*-values > 0.9320). This suggests that at these concentrations, the EOB did not significantly impact the fitness of *B. subtilis*. At 6.25% EOB, the relative fitness decreased slightly to RF = 0.61, but this reduction was not statistically significant (*p* = 0.9970). This indicates that this concentration began to have a minor inhibitory effect on *B. subtilis*, although it was not substantial. A significant decrease in fitness was observed at higher concentrations. At 12.5% EOB, the relative fitness decreased sharply to RF = 0.33 (*p* < 0.0001), indicating a strong inhibitory effect. This trend continued, with the fitness dropping further to RF = 0.10 at 25% EOB (*p* < 0.0001) and to an RF of nearly zero at the 50% EOB concentration. These findings suggest that high concentrations of the EOB are highly effective in inhibiting or killing *B. subtilis*.

### 3.4. Bacterial Growth Metrics

#### 3.4.1. Carrying Capacity (k)

**Figure 3:**
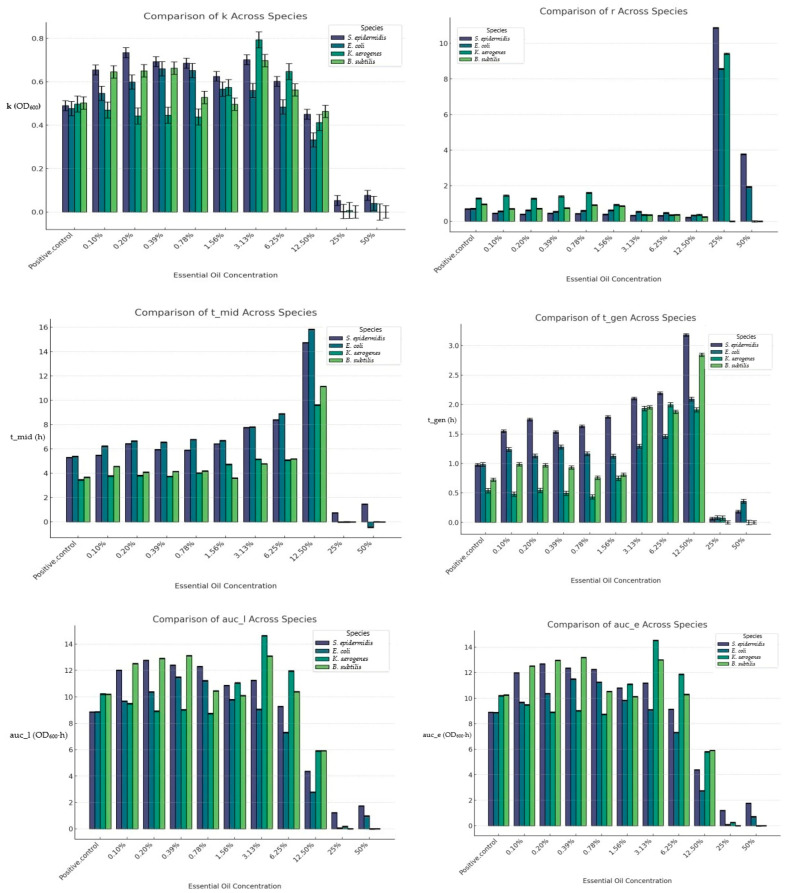
Effect of EOB on the carrying capacity, growth rate (r), time to mid-log exponential phase (t_mid), generation time (t_gen), area under the logistic curve (auc_l), and area under the empirical curve (auc_e) across all the bacterial species.

The carrying capacity, measured in OD_600_ units, is shown in Figure 3. The values for this parameter were inconsistent across the bacterial species at lower and intermediate concentrations but were generally higher than those for the control. There was a notable decline at 12.5% EOB and a drastic decline at 25 and 50% EOB.

#### 3.4.2. Growth Rate (r)

The growth rate data, expressed in h^−1^, are presented in Figure 3. *Staphylococcus* exhibited a reduced growth rate from the control to 12.50% EOB but showed increased growth rates at 25 and 50% EOB. *E. coli* displayed a similar pattern, with its growth rate generally decreasing from the control to the 12.50% EOB. *K. aerogenes* showed inconsistent trends from the control at EOB concentrations up to 0.78% and a marked decline from 1.56 to 12.50% EOB but there was a sharp increase at 25% and a sharp decrease again to zero at 50% EOB. *Bacillus* also had inconsistent trends up to 1.56%, which then decreased up to the 12.50% concentration and was down to zero at 25% EOB, indicating complete inhibition at the two highest concentrations.

#### 3.4.3. Time at Midpoint of the Growth Curve (t_mid)

Across all the bacterial species investigated, the t_mid values were lower for the control up to 12.5% EOB, indicating slower growth at these concentrations within this range (Figure 3). *E. coli* had the longest t_mid, indicating more delayed growth compared with the other bacteria. The highest values for t_mid across all the bacterial species were at the 12.5% EOB concentration, but these were sharply reduced at 25 and 50% EOB.

#### 3.4.4. Generation Time (t_gen)

All the bacterial species had higher t_gen values than the control across all the concentrations up to 12.5% EOB and then exhibited a sharp decline at 25 and 50% EOB (Figure 3). At all the concentrations up to 12.5% EOB, *Staphylococcus* had the highest value for t_gen.

#### 3.4.5. Area Under the Empirical (auc_e) and the Logistic (auc_l) Curve

The trends for auc_l and auc_e (OD_600_·h) are presented in Figure 3. The auc_l values across the bacterial species were generally higher than those for the control at the lower and intermediate concentrations. Unlike the observations for t_mid and t_gen, there was a sharp decline in auc_l at 12.5% EOB across all the bacterial species for this value before dropping to zero or near zero across the bacterial species at 25 and 50% EOB. The same trend was maintained for auc_e.

## 4. Discussion

### 4.1. Growth Curves

The growth curves illustrate the impact of the EOB on the growth dynamics of various bacterial species. The lag phase, characterized by low absorbance values, was generally observed within the first 2 h across all the EOB concentrations and bacterial species except at the sub-MIC concentration (12.5% EOB), where the lag phase extended up to 4 h. This extended lag phase suggests that the bacteria required more time to adapt to a sub-lethal concentration of the EOB, which aligns with findings from previous studies where sub-inhibitory concentrations of carvacrol prolonged the lag phase of bacteria [21]. Maggio et al. [22] reported an extended lag phase with exposure to *Origanum vulgare* EO at a sublethal concentration.

Between 4 to 10 h, there was a significant increase in absorbance in the control (0% EOB concentration) and when lower concentrations of the EOB (0.1 to 0.78% EOB) were used, indicating rapid bacterial growth. This growth pattern is consistent with the exponential phase, where bacteria multiply rapidly due to the availability of nutrients and the relatively low stress imposed by these lower concentrations of the EOB. This observation is similar to findings by Lambert et al. [23], who reported that lower concentrations of essential oils allowed for normal bacterial growth rates, with minimal disruption to cellular functions. The intermediate concentrations (1.56 to 3.13% EOB) produced inconsistent trends in the absorbance, suggesting variable effects of the EOB at these concentrations. Such variability might be due to the partial inhibition of some bacterial cells, leading to fluctuations in growth as the bacteria attempt to overcome the antimicrobial effects. This is consistent with a previous report by Bassolé and Juliani [24], who suggested that essential oils can cause dose-dependent inhibition of bacterial growth and require relatively higher concentrations to exert their antimicrobial potentials.

At higher concentrations (25 and 50% EOB), the growth curves were flat across all the bacterial species, indicating little to no growth. This complete inhibition at high concentrations is in line with previous studies by Zainal-Abidin et al. [25] and Wang et al. [26], who demonstrated that essential oils at sufficient concentrations can entirely inhibit bacterial growth by causing irreversible damage to the bacterial cell membrane and interfering with key metabolic processes.

The sub-MIC concentration (12.5% EOB) produced a weak increase in absorbance across all bacterial species, indicating limited bacterial growth. This sub-lethal concentration likely allowed for some bacterial survival, but the EOB’s inhibitory effects were strong enough to prevent robust growth. This observation is consistent with the findings of Siroli et al. [27], where thyme and oregano EOs and their bioactive compounds led to a decrease in the growth rate during the exponential phase and an increase in the lag phase.

### 4.2. Relative Fitness

The relative fitness across the bacterial species demonstrated that the EOB exerted a concentration-dependent inhibitory effect. The fitness of the bacterial cells remained unaffected at lower concentrations (0.1 to 3.13% EOB), indicating that these concentrations were not sufficient to exert a significant antimicrobial effect. This lack of a significant effect at lower concentrations is consistent with the findings of Sharifi-Rad et al. [28], who reported that low concentrations of garlic oil exhibited minimal antimicrobial activity against *S. aureus*. Similarly, Saeed and Tariq [29] observed that while EOs were effective against *S. aureus*, the concentration had to exceed a specific threshold to achieve significant bactericidal effects. A noticeable decline in bacterial fitness began at the 6.25% concentration, where the fitness decreased by nearly 40%, suggesting that the EOB began to affect bacterial viability or replication capacity at this threshold.

### 4.3. Bacterial Growth Characteristics

The reductions in the carrying capacity and growth rate suggest that the EOB exerted a measurable inhibitory effect on all the bacterial species. The carrying capacity is the largest population size that a given environment can support indefinitely [30]. It is the greatest number of cells that can be supported by the available nutrients, space, and overall conditions [31]. The growth rate measures how quickly the bacterial population increases during the exponential phase of growth and is calculated as the rate of change of the population size per unit time, with higher values indicating faster growth [32].

The increase in generation time (t_gen) and the delay in reaching the midpoint of the growth curve (t_mid) suggest that the EOB used in this study can interfere with bacterial replication. Generation time is defined as the time required for a bacterial population to double in number and acts as an important measure of the growth rate of bacteria, reflecting how quickly a population can expand under given conditions [33]. A shorter generation time signifies a faster growth rate, indicating efficient reproduction under the given conditions. The generation time varies depending on the species and the environmental conditions, with optimal conditions leading to shorter generation times, and can be a critical indicator of bacterial adaptation or resistance [34]. The highest value for this parameter was recorded for *S. epidermidis*, indicating that it was more sensitive than the other bacterial species used in the present study. This is similar to the trend observed in the study by Aćimović et al. [35], where *S. aureus* was more sensitive than the other bacterial species investigated across all the tested EOBs, while *K. aerogenes* had the lowest value, indicating that it was the least susceptible.

The time to the mid-log exponential phase (tmid) increased with higher concentrations of EOs across most of the bacterial species, with the longest tmid observed at the highest sub-lethal concentrations (12.5% EOB) across the bacterial species. Essential oils are known to disrupt bacterial membrane integrity, leading to increased permeability and the loss of cellular components [36]. This disruption can trigger a longer lag phase, as cells must expend energy to restore membrane integrity and to prevent further leakage of vital intracellular components, thus reflecting the time needed to repair membrane damage and re-establish homeostasis [37].

The observed reduction in bacterial biomass (auc_l and auc_e), especially at higher concentrations, indicates that EOs could effectively reduce the bacterial load, for example, in clinical settings, potentially mitigating the severity of infections and reducing the spread of resistant strains. The area under the logistic curve (auc_l), based on the logistic growth model, quantifies the overall growth of the bacterial population over time. It integrates both the growth rate and the carrying capacity, offering a comprehensive measure of bacterial productivity. In contrast, the area under the empirical curve (auc_e) represents the total observed growth of the bacterial population over time and is based on empirical data. It is used to assess the accuracy of growth models and to account for deviations from expected growth patterns [38]. Both parameters are proxies for bacterial biomass and have a relationship with the carrying capacity and the generation time [38].

The antimicrobial efficacy of the EOB against the bacterial species can be attributed to its diverse bioactive constituents and their specific modes of action. Monoterpene alcohols and phenolic derivatives in the EOB are among the most potent antibacterial agents. Notably, most of the bioactive compounds in the EOB are known to compromise bacterial membrane integrity (Terpinen-4-ol, p-cymen-7-ol, eucalyptol, β-Caryophyllene oxide). Terpinen-4-ol is well known for its broad-spectrum bactericidal activity and disrupts both microbial cell wall and membranes, leading to leakage of cytoplasmic contents such as Ca^2^^+^ and Mg^2^^+^ ions as well as lactate dehydrogenase [39]. These compounds can induce oxidative stress inside bacterial cells—for example, cymen-7-ol and trace phenolics like methyleugenol have been shown to trigger intracellular reactive oxygen species (ROS) generation—contributing to cell damage [40,41]. Eucalyptol and β-Caryophyllene oxide have moderate direct bactericidal potency compared with phenolics, but their membrane-permeabilizing action may facilitate the uptake of other bioactive molecules; hence, they may act synergistically and extend the spectrum of the EOB’s activity [42]. Organosulfur compounds from the garlic fraction of the EOB could impart a different mode of antibacterial action. Allyl methyl sulfide is a stable decomposition product of allicin and can interfere with bacterial metabolism by inactivating essential enzymes (e.g., cysteine proteases, dehydrogenases) and disturbs the cellular redox balance, thereby inducing a state of thiol stress and oxidative damage in the microbe [43]. The combined presence of these multifaceted compounds likely underlies the potent antimicrobial activity observed for this EOB. Each constituent may target different cellular structures or processes so, together, they could deliver a broad-spectrum, multi-target assault on bacterial cells. For example, membrane-disrupting terpenes can enhance the uptake of other molecules. Such synergistic interactions among the EOB components may explain why a blend of essential oils exhibits strong bactericidal effects even when some individual constituents are only moderately active. The temporary increases in fitness encountered in the present study with the introduction of the EOB could be attributed to hormesis. This biological phenomenon occurs when a lower dose of a potentially harmful agent induces a beneficial effect in the organism, such as enhanced growth activity or survival, while higher doses of the same agent may be detrimental or even lethal [44]. Exposure to EOs at sub-inhibitory concentrations can induce stress responses in bacteria, which may temporarily enhance their fitness or resistance [45].

## 5. Conclusions

This study presents new information on the mode of antimicrobial action of a uniquely formulated EOB and its ability to inhibit bacterial growth on a concentration-dependent basis via several mechanisms of action. The results revealed that higher concentrations of the EOB completely inhibited bacterial growth. Furthermore, the study demonstrated that the sub-MIC concentration significantly extended the lag phase and weakened the exponential growth, suggesting a potent adaptive challenge for bacteria even below inhibitory thresholds. The clinical relevance of the results is significant, particularly in the context of antibiotic resistance and the overdependence on synthetic antibiotics. The broad-spectrum antimicrobial activity of the EOB shows potential for it to serve as an alternative or adjunct therapy to traditional antibiotics. However, further studies should investigate a broader range of pathogenic bacteria and subject the organisms to sub-MICs of the EOB for an extended period to test for resistance. We also recommend investigating the gene expression profiles of these bacterial species affected by the EOB. Overall, there is a need to explore the in vivo efficacy, safety, and potential synergistic effects of the EOB when used alone or in combination with conventional antibiotics.

## Figures and Tables

**Table 1:** Volatile compounds in the essential oil blend identified by GC-MS analysis.

Compound Name	RT ^1^	Formula	Concentration (%) ^2^
2-Propanamine, 2-methyl-	8.78	C_4_H_11_N	1.13
Sulfide, allyl methyl	9.43	C_4_H_8_S	2.03
Bicyclo[2.2.1]heptane, 2,2-dimethyl-3-methylene-, (1S)-	10.06	C_10_H_16_	6.77
Beta-Phellandrene	10.87	C_10_H_16_	20.3
Beta-Myrcene	11.14	C_10_H_16_	12.1
1,3-Cyclohexadiene, 1-methyl-4-(1-methylethyl)-	12.75	C_10_H_16_	6.45
Eucalyptol	12.86	C_10_H_18_O	6.24
4-Nonanone	15.43	C_9_H_18_O	5.18
5-Hepten-2-one, 6-methyl-	15.7	C_8_H_14_O	3.00
Benzene, 1-methyl-3-(1-methylethenyl)-	16.18	C_10_H_12_	2.93
Bicyclo[3.1.0]hexan-2-ol, 2-methyl-5-(1-methylethyl)-, (1α,2α,5α)-	16.75	C_10_H_18_O	1.91
Bicyclo[7.2.0]undec-4-ene, 4,11,11-trimethyl-8-methylene-,[1R-(1R*,4Z,9S*)]-	17.67	C_15_H_24_	0.87
Bicyclo[3.1.0]hexan-2-ol, 2-methyl-5-(1-methylethyl)-, (1α,2α,5α)-	18.13	C_10_H_18_O	1.25
Terpinen-4-ol	18.67	C_10_H_18_O	10.53
Geranyl formate	19.5	C_11_H_18_O_2_	9.36
3-Furaldehyde	22.0	C_5_H_4_O_2_	0.46
Methyleugenol	24.39	C_11_H_14_O_2_	1.62
Caryophyllene oxide	24.68	C_15_H_24_O	1.13
p-Cymen-7-ol	25.27	C_10_H_14_O	6.71

^1^ RT, retention time (min). ^2^ Concentration based on the total area of the identified peaks.

**Table 2:** Relative fitness of *S. epidermidis*, *E. coli*, *K. aerogenes*, and *B. subtilis* in response to EOB concentrations.

Assess Details	Mean 0% (Positive Control)	Mean Concentrations	Standard Error	Adjusted *p*-Value
***S. epidermidis***				
0% vs. 0.1%	0.8509	0.8512	0.06542	>0.9999
0% vs. 0.2%	0.8509	0.8491	0.06542	>0.9999
0% vs. 0.39%	0.8509	0.8354	0.06542	>0.9999
0% vs. 0.78%	0.8509	0.8248	0.06542	>0.9999
0% vs. 1.56%	0.8509	0.7180	0.06542	0.6275
0% vs. 3.13%	0.8509	0.6715	0.06542	0.1987
0% vs. 6.25%	0.8509	0.5104	0.06542	<0.0001
0% vs. 12.50%	0.8509	0.2137	0.06542	<0.0001
0% vs. 25%	0.8509	0.05653	0.06542	<0.0001
0% vs. 50%	0.8509	0.1495	0.06542	<0.0001
***E. coli***				
0% vs. 0.1%	0.7647	0.7861	0.07988	>0.9999
0% vs. 0.2%	0.7647	0.79	0.07988	>0.9999
0% vs. 0.39%	0.7647	0.8929	0.07988	0.8759
0% vs. 0.78%	0.7647	0.8262	0.07988	0.9995
0% vs. 1.56%	0.7647	0.732	0.07988	>0.9999
0% vs. 3.13%	0.7647	0.6288	0.07988	0.8313
0% vs. 6.25%	0.7647	0.4771	0.07988	0.0212
0% vs. 12.50%	0.7647	0.1551	0.07988	<0.0001
0% vs. 25%	0.7647	0.01499	0.07988	<0.0001
0% vs. 50%	0.7647	0.1863	0.07988	<0.0001
***K. aerogenes***				
0% vs. 0.1%	0.6912	0.6172	0.08149	0.9979
0% vs. 0.2%	0.6912	0.5767	0.08149	0.9440
0% vs. 0.39%	0.6912	0.5777	0.08149	0.9472
0% vs. 0.78%	0.6912	0.5652	0.08149	0.8995
0% vs. 1.56%	0.6912	0.6492	0.08149	>0.9999
0% vs. 3.13%	0.6912	0.8422	0.08149	0.745
0% vs. 6.25%	0.6912	0.7325	0.08149	>0.9999
0% vs. 12.50%	0.6912	0.317	0.08149	0.0007
0% vs. 25%	0.6912	0.07049	0.08149	<0.0001
0% vs. 50%	0.6912	0.3202	0.08149	0.0008
***B. subtilis***				
0% vs. 0.1%	0.6669	0.749	0.05662	0.9320
0% vs. 0.2%	0.6669	0.8207	0.05662	0.2098
0% vs. 0.39%	0.6669	0.8155	0.05662	0.2531
0% vs. 0.78%	0.6669	0.6575	0.05662	>0.9999
0% vs. 1.56%	0.6669	0.6666	0.05662	>0.9999
0% vs. 3.13%	0.6669	0.8017	0.05662	0.3913
0% vs. 6.25%	0.6669	0.6134	0.05662	0.997
0% vs. 12.50%	0.6669	0.3319	0.05662	<0.0001
0% vs. 25%	0.6669	0.1009	0.05662	<0.0001
0% vs. 50%	0.6669	0.000157	0.05662	<0.0001

## Data Availability

The original contributions presented in the study are included in the article; further inquiries can be directed to the corresponding authors.

## Abbreviations

The following abbreviations are used in this manuscript:

EOB	essential oil blend
MICs	minimum inhibitory concentrations
EOs	essential oils
MRSA	methicillin-resistant *Staphylococcus aureus*
RF	relative fitness
ROS	reactive oxygen species
